# Helical Milling of CFRP/Ti6Al4V Stacks Using Nano Fluid Based Minimum Quantity Lubrication (NF-MQL): Investigations on Process Performance and Hole Integrity

**DOI:** 10.3390/ma16020566

**Published:** 2023-01-06

**Authors:** Kiran Mughal, Mohammad Pervez Mughal, Muhammad Umar Farooq, Muhammad Qaiser Saleem, Rodolfo Haber Guerra

**Affiliations:** 1 Department of Industrial and Manufacturing Engineering, University of Engineering and Technology Lahore, Lahore 54890, Pakistan; 2 Department of Industrial Engineering, University of Management and Technology Lahore, Lahore, Pakistan; 3 School of Mechanical Engineering, University of Leeds, Leeds LS2 9JR, UK; 4 Center for Automation and Robotics (UPM-CSIC), Ctra. De Campo Real 0,200. La Poveda, 28500 Madrid, Spain

**Keywords:** CFRP, Ti6Al4V, milling, minimum quantity lubrication, sustainability

## Abstract

The structural components in the aeronautical industry require CFRP/Ti6Al4V stacks to be processed together, which results in poor hole integrity due to the thermal properties of the materials and challenges related to processability. These challenges include quality variation of the machined holes because of the limitations in process properties. Therefore, a novel solution through helical milling is investigated in the study using nano fluid based minimum quantity lubrication (NF-MQL). The analysis of variance shows, for Ti6Al4V, eccentricity (PCR = 28.56%), spindle speed (Ti) (PCR = 42.84%), and tangential feed (PCR = 8.61%), and for CFRP, tangential feed (PCR = 40.16%), spindle speed (PCR = 28.75%), and eccentricity (PCR = 8.41%) are the most significant parameters for diametric error. Further on, the rise in the circularity error is observed because of prolonged tool engagement at a higher value of tangential feed. Moreover, the surface roughness of Ti was reduced with an increasing percentage of MoS_2_ in the lubricant. The spindle speed (37.37%) and lubricant (45.76%) have a potential influence on the processing temperature, as evident in the analysis of variance. Similarly, spindle speed Ti (61.16%), tangential feed (23.37%), and lubrication (11.32%) controlled flank wear, which is critical to tool life. Moreover, the concentration of MoS_2_ decreased edge wear from ~105 µm (0.5% concentration) to ~70 µm (1% concentration). Thorough analyses on process performance in terms of hole accuracy, surface roughness, processing temperature, and tool wear are carried out based on the physical science of the process for cleaner production. The NF-MQL has significantly improved process performance and hole integrity.

## 1. Introduction

Carbon fibre-reinforced polymer (CFRP) is extensively used in a variety of industries because of its high strength-weight ratio and remarkable corrosion resistance [1]. In the aerospace industry, CFRP components are assembled with Ti alloy components to form CFRP/Ti stacks. In this regard, the stacking of CFRP with Ti requires drilling holes in the stacks as the components are typically riveted or bolted [2]. It is preferred that the stacks be machined in conjunction, despite machining them separately to achieve greater geometric accuracy. Hole drilling in stacks is particularly challenging due to the disparity in mechanical properties between CFRP and Ti. Due to such reasons, an explanation of the hole-making process of CFRP/Ti stacks and improving the quality of results have received considerable attention from the researchers.

Based on general milling principles, helical milling is a distinct hole-making process that makes milling tools travel on a spiral path. By changing the eccentricity (the distance between the hole axis and the tool centre) holes of different diameters can be milled without changing the tool. The process brings down processing costs and enhances processing efficiency [3]. Compared with conventional drilling procedures [4,5], helical milling can produce holes with a higher fatigue life and better quality. Helical milling is cost-effective because it can produce different sized holes with one tool by changing eccentricity, has higher processing efficiency than conventional drilling [6], and lower tool wear [7]. It is noted in the research literature that helical milling could save up to 30% in manufacturing costs for various aircraft parts. Apart from producing holes of different diameters, varying eccentricity values affect the performance measures. Larger values of eccentricity not only provide improved chip removal and heat dissipation through radial clearance but also decrease the thrust force required for machining. The burr formation at the entry and exit of the hole becomes a severe matter of concern [8]. The formation of burrs are aggravated with increasing thrust force and tool wear [9]. Similarly, delamination is among the foremost obstacles to machining CFRP.

During machining, CFRP/Ti stacks can withstand the processing damage, which affects the quality of the holes being drilled. He et al. [10] compared the effect of constant parameters with variable helical milling on cutting force and hole quality at entry and exit. After deductions were made, it was found that the variable parameters of the machining enhanced the geometric accuracy of the holes. Hrechuk et al. [11] developed hole damage criteria for hole-integrity evaluation based on discernible damages, i.e., uncut fibre and delamination. They also inferred a linear relationship between hole quality, the number of holes drilled, and tool wear. Li et al. [12] examined hole quality and tool wear in dry helical milling of Ti-6Al-4V and found that surface roughness was diminished near the end of tool life due to microsmearing. Since the hole quality during helical milling of stacked material is affected by different factors, surface roughness alone is not an appropriate measure to report hole quality, as microsmearing covers many sub-surface defects. Hole quality must be evaluated by measuring burr formation, circularity error, diametric error, and surface roughness. Moreover, the performance of the helical milling process is also limited by excess temperature generation, which deteriorates the service life of tooling and results in poor surface integrity due to aggressive cutting and recutting of chips.

Tool wear is a more serious problem in stack drilling than in single-layer drilling of CFRP or Ti. Tool wear is influenced by cutting temperature, machining parameters, stack sequence, tool geometry, and cooling conditions [13]. A comparison of single-layer machining of CFRP and Ti with that of CFRP/Ti stacks was conducted by Wang et al. [14]. They concluded that during the machining procedures for Ti, the dominant wear mechanisms are chipping of the tool and adhesive wear. While in the case of CFRP, abrasive wear turned out to be the dominant wear mechanism. In the case of helical milling of CFRP/Ti stacks, tool wear occurs by both of the abovementioned mechanisms. Xu et al. [15] analysed the hole quality of holes made through the helical milling of CFRP/Ti stacks and found that tool wear markedly affected the hole quality. The study also recommended using a retraction cooling process to reduce the cutting temperature, thereby reducing tool wear. High cutting temperatures significantly affect tool wear. It influences the thermal-mechanical behaviour of CFRP/Ti stacks, which can cause some thermal degradation to CFRP during helical milling of the stacks [16].

Furthermore, Wang et al. [17] reported debonding and resin degradation in the CFRP layer as the cutting temperature rose above the glass transition temperature of the resin. Therefore, using cooling techniques to keep the temperature under control is of prime importance. The preeminent cooling techniques in machining are flood cooling, minimum quantity lubrication (MQL), and cryogenic cooling. MQL has the potential to reduce the cutting temperature and vary thermal-mechanical behaviour by truncating the friction coefficient between workpiece and tool [18]. MQL is widely implemented in machining various difficult-to-mine alloys, as it greatly reduces cutting temperature and thrust forces and improves surface integrity [19]. Sasahara et al. [20] compared helical milling of aluminium with conventional drilling under MQL and dry conditions. Sasahara et al. observed smaller cutting forces, lower cutting temperatures, lower burr heights, and better hole quality under MQL conditions compared to dry conditions. Ge et al. [21] collocated MQL with dry and cryogenic conditions in helical milling of CFRP/Ti stacks and observed extrusion fracture in the fibres of CFRP and reduced feed marks at the Ti surface. They also reported a considerable reduction in circularity errors under MQL conditions as compared to those under dry and cryogenic conditions. Similar effects were reported [18] for drilling the of CFRP/Ti stacks under MQL conditions. Tool wear under MQL conditions was reduced as compared to dry and cryogenic conditions. Qin et al. [22] observed reduced surface roughness and tool wear during helical milling of Ti-6Al-4V alloy under MQL conditions. Xu et al. [23] reported improved surface morphologies and reduced tool wear in the drilling of CFRP/Ti stacks under MQL conditions. The results recommended that MQL produced thrust forces in drilling, delamination, and geometric accuracy comparable to that of conventional dry drilling. Xu et al. [24] reported improved machining qualities by using MQL in the drilling of CFRP/Ti stacks. They also observed slighter burr formation in Ti, improved surface morphology in CFRP, and enhanced quality in the drilled holes. It is clear from the above discussion that MQL produces better outcomes in terms of tool wear and comparable results with other lubrication systems in terms of thrust forces and hole quality. This further demonstrates that flood cooling can be substituted with MQL cooling because MQL uses less lubricant, reduces costs, and protects the environment.

In order to further improve the efficiency of MQL, researchers have proposed a new, more clean, low carbon, and low consumption technique named nano fluid minimum quantity lubrication (NF-MQL). NF-MQL carries enhanced tribological characteristics and a higher heat transfer capability than simple MQL technology. In NF-MQL, nanoparticles with various physical and chemical properties are first mixed with the MQL base oil, then atomised under the influence of high-pressure airflow, and then sprayed on the cutting region through the nozzle [25]. Multiple researchers have studied the effect of MQL and NF-MQL on the elevation of temperature, chip formation, and flank wear of the tool during machining of different hard-to-cut materials and CFRP/Ti6Al4V stacks. Jadam et al. [26] studied the impact of multi-walled carbon nano-tubes (MWCNTs) and added rice bran oil under MQL conditions during turning of a Ti–6Al–4V workpiece using TTS (P25) uncoated straight tungsten carbide inserts having attached chip breaker mechanisms on the evolution of cutting forces and tool-tip temperature, flak wear, and workpiece roughness. It was concluded that the use of NF-MQL during the process of turning Ti6Al4V can result in up to a 62.7% reduction in tool-tip temperature, an 8.69% reduction in the tangential component of the cutting force, a 42% reduction in the depth of flank wear, and a 12.8% reduction in the surface roughness of the product when compared to dry machining. Similarly, Duc et al. [27] evaluated the performance of minimum quantity lubrication (MQL) and minimum quantity cooling lubrication (MQCL) during hard drilling of Hardox 500 steel (49–50 HRC) using an Al_2_O_3_ nanofluid mixed in a water-based emulsion and rice bran oil with the help of coated carbide drills. The detailed chip morphology study revealed that MQCL using an Al_2_O_3_ nanofluid had better results and a significant reduction in the flank wear of the tool. Moreover, a significant decrease in surface roughness and machining temperature was reported during both MQL and MQCL conditions. Liu et al. [28] examined the effect of dry, MQL, and high-pressured air cooling/lubrication conditions on tool life and surface finish of Ti6Al4V during the end milling operation. The experimentation process was conducted at two different cutting speeds (60 m/min and 150 m/min), and it was observed that both MQL and high-pressure air conditions can reduce the friction pressure coefficient (Kf) drastically (up to 50%) and hence reduce the tool-tip temperature significantly. It was also concluded that MQL has the ability to reduce friction at the chip-tool interface, making it a favourable green approach to machining difficult-to-cut material. In another study [29], the cooling performance of nanoparticles including Al_2_O_3_, MoS_2_, SiO_2_, CNTs, SiC, and graphite particles was analysed using the Bap300r-c16-160-160l milling tool bar with an APMT1135PEDR blade on the Ti-6Al-4V. The researchers reported that Al_2_O_3_ nanoparticles can effectively decrease the milling force and that SiO_2_ nano fluid/particles have the best cooling capacity among the selected nano fluid/particles. They also reported that Al_2_O_3_ and SiO_2_ nanoparticles were the best nanoparticles for the MQL condition by providing low surface roughness values. Moreover, these particles are the most suitable environmentally friendly additive nanoparticles for the base oil. The characteristics of Al_2_O_3_ and SiO_2_ nanoparticles contribute to the long life of the tool and the workpiece. Hegab et al. [30] investigated the effect of MQL-MWCNT (multi-walled carbon nanotubes) and aluminium oxide (Al_2_O_3_) gamma nanoparticles during the turning of Inconel 718 with a tungsten carbide cutting insert. Tool performance and chip morphology were thoroughly studied, and it was concluded that both nano fluids showed promising cutting tool performance with minimum flank wear, notching, build-up edge, and oxidation. Moreover, a significant decrease in tool temperature was noticed when the NF-MQL condition was employed.

Moreover, it is obvious from the literature presented above that most of the studies are engrossed in the effects of eccentricity on the helical milling of Ti and its alloys. Correspondingly, MQL has been implemented in the helical milling of Ti, its alloys, and the drilling and milling of CFRP/Ti stacks. But its effects, by using novel nano fluids on machining characteristics of CFRP/Ti stacks in case of helical milling, are not well investigated yet. This study fills this aperture and investigates the effects of eccentricity and MQL deploying MoS_2_ on the surface integrity, machining temperature and tool wear in helical milling of CFRP/Ti stacks.

The objectives of the study are as follows:

- To optimise the process parameters in helical milling of CFRP/Ti-6AL-4V stacks to achieve better machining quality and minimum tool wear.
- To optimise the spray and machining parameters and minimise the tool wear and surface integrity damage.
- To evaluate machining performance in tool wear, hole accuracy, and surface integrity.

## 2. Experimental Setup

In this investigation, 200 g/m^2^ of 31-layered 3K carbon fibre with a 0°/90° orientation was used with a bisphenolic-based Vinyl Ester Resin Epovia RF-1001 matrix to make a 230 × 230 × 8 mm^3^ multidirectional quasi-isotropic [0°/90°/+45°/−45°] specimen of CFRP by using the hand lay-up method. The mechanical properties of T300 carbon fibre are shown in Table 1.

The tool’s geometry was selected from the literature to minimise the damage to holes. A 4-fluted 6 mm Firex coated end mill made by Guhring was used with a 30° Helix angle, and aluminium-2024 was used as a backup plate. In addition, tool overhang length was maintained at 40 mm as per literature on high-speed milling to avoid tool flattering and chattering [32]. Taguchi design of experiment was used, considering the cost and time, and the design matrix is provided in Table 2. An analysis of variance was carried out to determine the parametric influence on the process.

**Figure 1:**
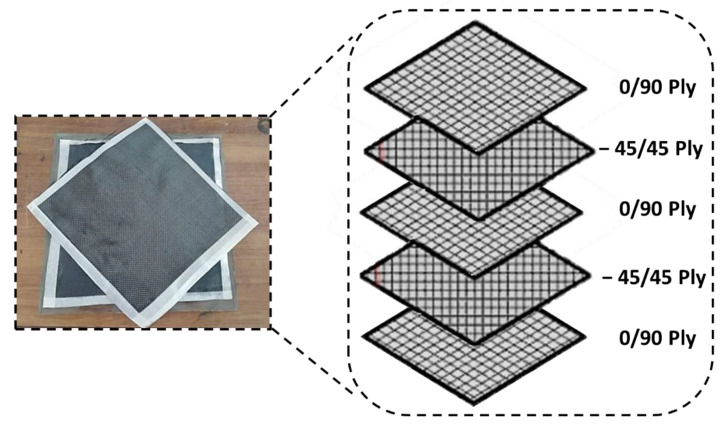
Multidirectional quasi-isotropic [0/90/+45/−45] specimen.

The material lay-up is schematically illustrated in Figure 1, based on the learnings from Hynes et al. [1]. A drying oven was used to post-cure the CFRP stack at 120 °C for 1 h. Titanium is kept on the upper side of the stack. The aluminium 2024 plate (13 mm thick) is used as a backup/supporting plate for CFRP on the bottom side. The plate is mechanically fastened (nut and bolt) using a micro-torque wrench with a torque of 34 Nm uniformly applied to all four corners. The uniformity of torque distribution is ensured at all points where nuts and bolts have been tightened. Therefore, a standard procedure related to axle offset was ensured before each experiment. The supporting plate reduces the vibrations, eliminates the chattering of tools, extrudes, and absorbs heat (heat sink) at the end of the holes produced by the continually proceeding helical milling process.

**Figure 2:**
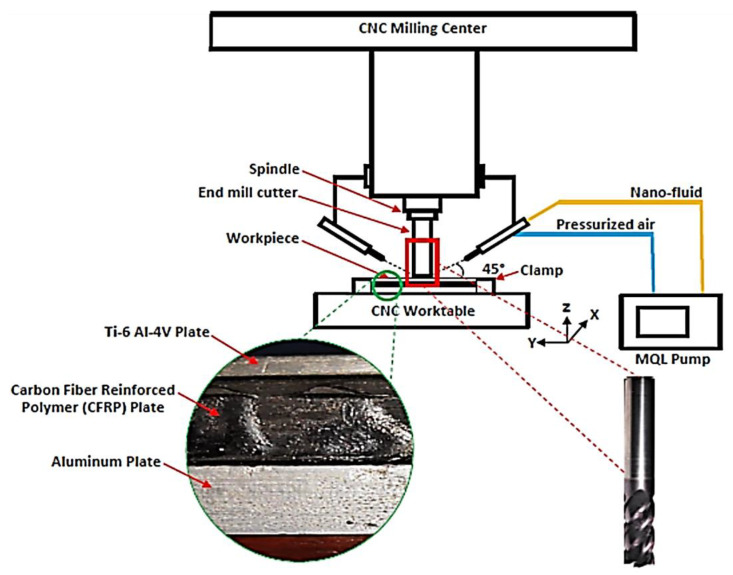
Process schematic for helical milling of CFRP/Ti stacks.

For the experimentation, an MCV-600 vertical milling centre by Long Chang Machinery Co. Ltd. was employed with a separate MQL setup. Helical milling is performed on a CFRP/Ti-6Al-4V workpiece using 6 mm-diameter Firex-coated end mills. MoS_2_-based minimum quantity lubrication is used to cut Ti-6Al-4V with a pressure of 4 bars, and a flow rate of 40 mL/hr. CFRP is machined dry with a continuous high pressure air supply. The complete machining process of the Ti/CFRP stack was completed in three rounds, from the first layer of titanium to the last layer of aluminium in the stack. In the first round, Stack was machined down to titanium only, and the tool was retracted back. In the second round, the stack was machined down to the complete thickness of the CFRP layer and retracted back. The tool was descended in the third phase to ensure CFRP was through-holed. The rationale behind the above machining scheme was to add a short temperature break interval between the first and the second phase, ensure easy burr/dust (if any) evacuation after tool retraction. The process schematic shows a detailed experimental setup in Figure 2, including the cutting tool, MQL setup, and workpiece.

**Figure 3:**
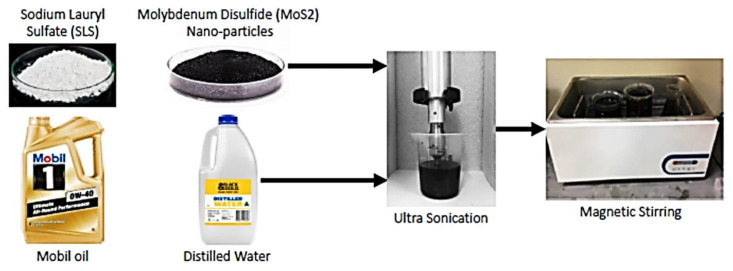
Nano fluid preparation mechanism.

The lubricant was prepared with 80% distilled water, 19% Mobil Oil Gold (Grade OW-40 fully synthetic), and 5 g Sodium dodecyl sulphate (as surfactant), with the addition of 0.5%, 0.75%, and 1% of solid lubricant MoS_2_ as per the design matrix (Figure 3).

It was recommended to keep the titanium at the top and CFRP at the bottom during machining. The reasons include keeping the surface well lubricated during the machining of titanium, and lubrication was off during the machining of CFRP. Moreover, the machining of CFRP is subjected to high pressure air to reduce abrasiveness to a minimum through instantaneous fibre dust removal, ensuring no compromise on the hole quality. High pressure air ensured the machining without burrs, dust particles, delamination, and uncut fibres. Minimum quantity lubrication helped ensuring fine machined holes with very minimal uncut fibres of CFRP as well as burrs of titanium. Ten holes were helically milled in each set of experiments, and performance measures were recorded as circularity error, diametric variation, maximum cutting temperature, and surface roughness. A coordinate measuring machine (CMM) (CE-450) by Chen Wei Precise Technology Co [33] was used to measure diametric variations. It has both contact and non-contact type options with 1 micron accuracy and 70x magnification. Quadra Check 500 (QC 500) supportable software was used for all measurements and calibrations. The measurements of the circularity error at the entrance, the centre, and at the exit of the holes were carried out in contact-type mode. A 3 mm diameter spherical probe was used for taking measurements at nine points with three different combinations, and every point was 120° apart. The average values of those combinations were considered for the analysis. Diametric error and circularity error were measured at entry and exit for the CFRP plate and on the bottom side for the Ti plate because of the small thickness of the Ti plate. Diameters were measured using three points, and then an average of those readings was taken. Similarly, after each experimental round, the flank wear was measured through CMM. On the flank face, five measurements were made on each experiment to have a wider understanding of the behaviour of the tool. The maximum value of tool wear was taken for results and analysis. The workpiece was cross-sectioned after each experiment to measure the roughness precisely. A surface texture meter (Surtronic S-128) by Taylor Hobson Precision UK was used to measure the surface roughness (Ra, as it is the most commonly used in industry) of holes [34]. The surface texture meter had a gauge range of 300 mm, a resolution of 0.01 mm, a traverse length of 0.25–25 mm, and a diamond stylus with a cut-off length of 0.80 mm and an evaluation length of 4 mm. The measurements were made at four different points from both sides of the holes as per standard, and an average was computed. The complete set of experiments is repeated three times to avoid any external variation, and the mean value is used for analysis. An analysis of variance (ANOVA) was performed on Minitab V29 to see how the parameters influenced both materials [35]. A t-test was performed on how the values at exit and entry differed to see the spread. ANOVA is used to study the effects of input parameters on response measurements, and every measurement was calculated by taking the average of five holes. Hole accuracy was measured in terms of diametric error and circularity error by using a CMM.

$$Diametric\;Error\;\left(D.E\right)=\left|D_{nominal}-D_{measured}\right|$$

$$Circularity\;error=D_{max}-D_{min}$$

A thermal imager (Testo 868) was employed for recording the temperature measurements at the workpiece-tool interface point [36]. Measurement duration was the number of seconds during which titanium plate was machined and the tool retracted. The thermal imager was calibrated with the help of a thermocouple data logger by taking known temperatures as reference points. The thermal imager was used in the study because a three-layer stack and variations in the thermal conductivity of materials limited the use of a thermocouple for obtaining the quantitative temperature measurements. To compare overall temperature generation in the experiment, qualitative temperature measurements were taken from the tool-workpiece interface point through placing the equipment at an in-house designed fixture. The tool and equipment setup kept equally unchanged and calibrated throughout the measurement process provided rationale for the variation in temperature values for different sets of input parameters for process optimization.

## 3. Results and Discussion

### 3.1. Hole Accuracy

#### 3.1.1. Diametric Error

In the aerospace industry, hole quality is determined by geometric accuracy. The application requires a high degree of accuracy to incorporate design-for-assembly requirements [37,38]. The entry and exit diameters of the stack materials are measured against five holes at each parameter. The selection and repetition of the experiments and measurements are based on the rationale of nullifying the external influences and minimising human and machine errors. The fluctuation of geometrical dimension is more apparent in the CFRP material hole at entry and exit positions. However, it is more stable on titanium. It is established that during the processing of laminate material, the hole-making mechanism changes, which decreases or increases the diameter. Therefore, the designs are characterised so that parametric conditions result in a minimal error and the final tolerances are within the prescribed specifications.

The reason for the changed dimensional conditions is that different machining properties of materials influence the cutting mechanism. Dimensional accuracy is further controlled by the tool type, cooling mechanism, and geometric design to produce boreholes within anticipated tolerances [32].

The borehole diameter in the CFRP layer, regardless of the feed rates, is mainly greater by around 15 microns than that in the titanium layer. The bore diameter in both CFRP and titanium layers reduces with the increase in axial feed from 1 to 2 mm and the process forces. Similarly, higher bore diameters are obtained in CFRP and titanium, increasing the tangential feed from 0.01 mm/tooth to 0.02 mm/tooth with fewer process forces due to tool deflection as the regular feed forces occur. The actual diameter (8 mm for eccentricity of 1 mm and 10 mm for eccentricity of 2 mm) is considered for machining instead of the programmed. The tool centre point is deflected by the normal feed forces in the direction of the bore hole centre point. These observations highlight the deduction that tool deflections majorly cause the diameter deviations in CFRP, as well as the observation that titanium is observed to have an interrelationship in a linear fashion between borehole diameter and feed normal forces [39].

The variation in the diameter magnitudes for the inlet and exit points of the hole in CFRP range from −20 μm to +10 μm and −30 μm to +10 μm, respectively, while the variation in the inlet and exit points of the hole for titanium range from −50 μm to −20 μm and −35 μm to −15 μm, respectively. Generally, during the helical milling of CFRP only, the holes produced are almost undersized, and the opposite is the case with the helical milling of titanium. Nevertheless, the machining of the stack is precisely in contrast to the individual helical milling of CFRP and titanium. Hole diameter variations specify several essential attributes, such as tool wear when processing the titanium [12]. Diameter error increases considerably with the hole size variations observed to be increasing in the outlet of CFRP and the inlet of Ti alloy. Hole diameter only varies when transitioning between two materials during machining. The hole diameter magnitude falls out of the IT7-IT9 tolerance (IT7 tolerance range specifies the tolerance of 15 microns for the hole size 6–10 mm, and IT9 specifies 36 microns tolerance for the same hole size). The borehole diameter in this study varied by 3-13 microns in Ti and 11.5 to 46.5 microns in CFRP stacks. Therefore, reaming is consistently required [40].

**Figure 4:**
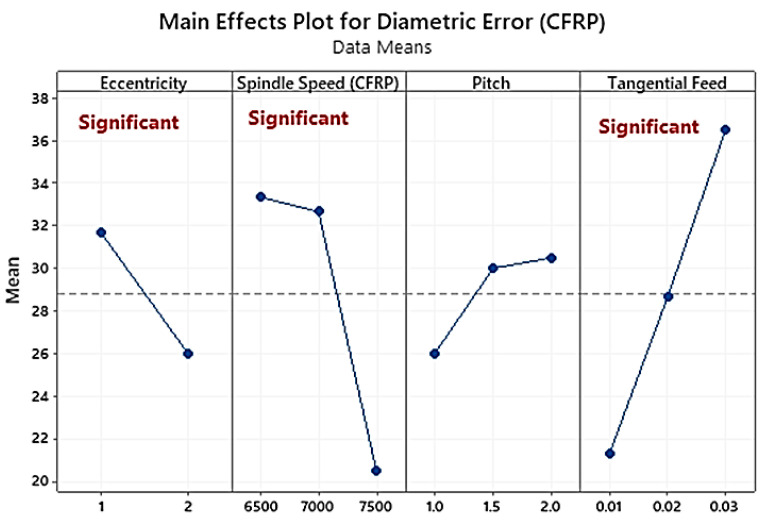
Main effects plot of diametric error in hole-making of CFRP.

For eccentricities of 1 and 2 mm, the diameters of helically milled holes in CFRP vary at 50 μm (Figure 4). The diameters of helically milled holes in Ti vary in the range of 48 μm for eccentricity of 1 mm and 30 μm for eccentricity of 2 mm. Conclusively, with an increase in eccentricity, the dimetric error decreases by 37.5% due to the provision of control over them. Similarly, the spindle speed used in this study (1000 to 1500 rpm for Ti) directly corresponds to the decrease in diametric error. However, the tangential feed is the opposite. The increase in the tangential feed from 0.01 mm/tooth to 0.03 mm/tooth translates into a higher dimensional error and almost doubles it from 4.8 microns to 8.8 microns. Against a 95% confidence interval, the analysis of variance (Table 3) shows that the significant factors in controlling diameters are tangential feed (PCR = 40.16%), spindle speed (PCR = 28.75%), and eccentricity (PCR = 8.41%), which are in agreement with the existing literature [23,40].

**Figure 5:**
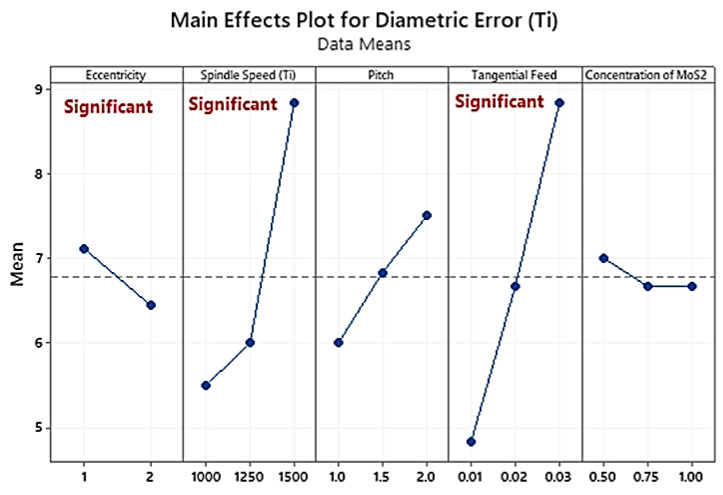
Main effects plot of diametric error in Ti6Al4V hole-making.

The holes produced in the CFRP layer are oversized. The compact carbon fibres cannot be machined at a lower cutting speed without unfavourable vibrations due to high workpiece and tool vibrations while machining CFRP at the low cutting speed [41]. The effect of eccentricity and tangential feed-in in the Ti layer is the same as in the CFRP layer (Figure 5). However, the error started increasing with the increase in the spindle speed from 6500 rpm to 7500 rpm, due to an unstable machining mechanism at a higher speed. The analysis of variance (Table 4) shows eccentricity (PCR = 28.56%), spindle speed (Ti) (PCR = 42.84%), and tangential feed (PCR = 8.61%) as the most significant parameters.

#### 3.1.2. Circularity Error

**Figure 6:**
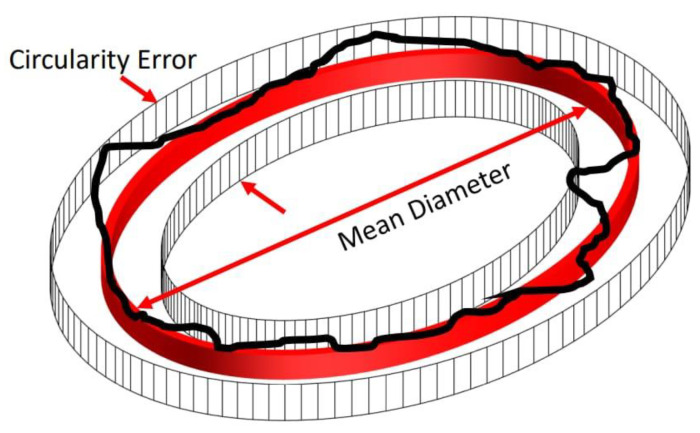
Circularity error.

Circularity error is the radial distance between the minimum circumscribing circle and the maximum inscribing circle, as shown in Figure 6 [42,43]. The quality of the hole in the case of Ti alloy remains good, while in the case of CFRP, delamination at the inlet is observed during the milling process [44]. The supporting plate benefits from standardising the outlet hole quality. The glitches in count and magnitude increase gradually in the inlet or outlet segments of CFRP, despite being on a single plate during the helical milling of the stack. The burrs in the inlet segment show a uniform rolled state about the titanium alloy, which gives rise to the deburring. In contrast, the burrs at the outlet are more significant in size and without a uniform distribution state. A similar phenomenon is observed by [39,45].

**Figure 7:**
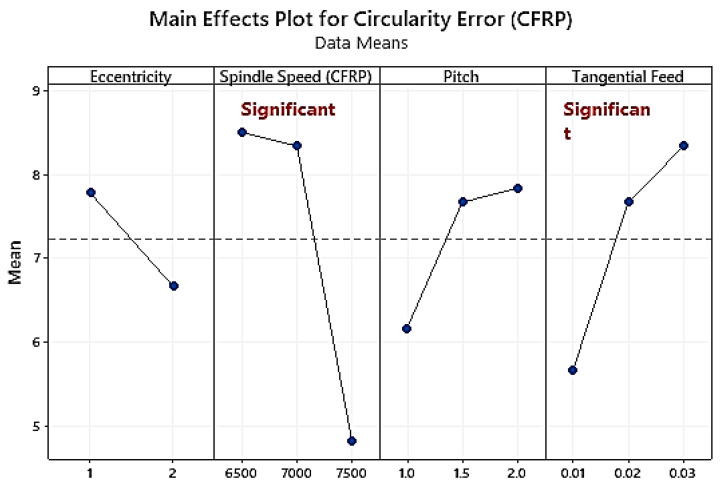
Main effects plot of circularity error in CFRP hole-making.

A t-test conducted on the circularity error at that entry and existing points of the holes has shown that there is no statistically significant difference in the values of the circularity error (t-value = 0.31, *p*-value = 0.758). The increase in the circularity error is caused by an increase in cutting temperature due to prolonged tool engagement at a higher value of tangential feed (Figure 7). At a lower feed rate (PCR = 16.6% for CFRP; PCR = 43.99% for Ti), the engagement time increases (Table 5 and Table 6). Using carbide tools to machine carbon fibre and epoxy matrix results in a lower circularity error because the thermal coefficient of the epoxy matrix and carbon fibre is three times lower than that of the carbide, which provides the tool with more stability and tempers the fibre to be penetrated easily. In the same way, Ashrafi et al. [46] concluded that radial and axial forces rise with higher feed values, resulting in deterioration of the end mill and higher circularity error. The spindle speed and tangential feed are dominant parameters, as evident through the analysis of CFRP variance (Table 5) and Ti6Al4V (Table 6).

Spindle speed (PCR = 30.55%) is an influential parameter in Ti layer hole-making (Table 6). It is another significant factor in machining that improves the tool’s rotational stability and reduces friction [16,40]. An increase in cutting speed (PCR = 29.51%) causes an increase in temperature, leading to the softening of fibre and ease in machining, thus minimising the circularity error for CFRP.

**Figure 8:**
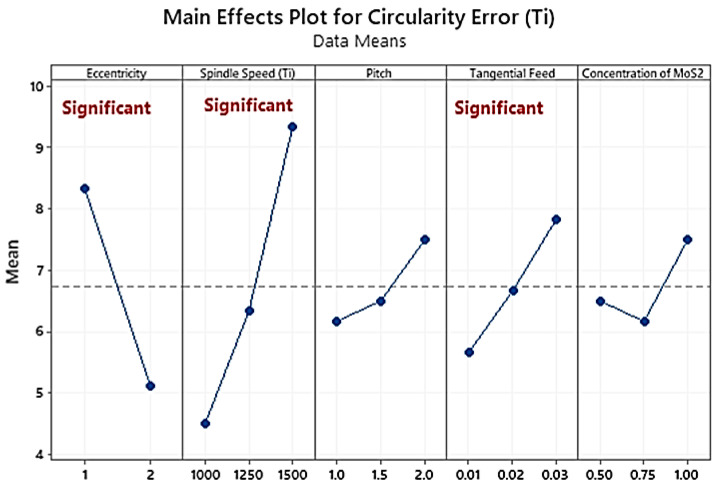
Main effects plot of circularity error in Ti6Al4V hole-making.

In the case of Ti (Figure 8), the circularity has doubled (from 4.5 microns to 9.333 microns) with the increasing spindle speed from 1000 rpm to 1500 rpm due to the rising temperature, which causes resin heating and hence the accuracy of the hole increases. A higher feed rate increases the thrust force, so the tool’s instability increases by leaving less accurate holes.

### 3.2. Surface Roughness

Obtaining hole quality in the machining of laminate is challenging due to the different materials. Another challenge is that the quality changes over time as tools wear out. Similarly, delamination at the entry is less because of minor wear, and it increases at the exit point due to impact on the tool during shifting to other materials. In this study, the surface roughness of the holes is measured at different points, and an average of the values is computed [47,48].

As evident from the analysis of variance (Table 7), spindle speed (PCR = 31.37%) and eccentricity (PCR = 7.32%) are the most influential parameters in controlling the surface roughness of CFRP. On the other hand, the traces of ablation at the joint indicate compromised surface quality. Similarly, the burr characteristics in the laminates at the entrance and exit are directly related to the tool wear that influences surface quality. The relation of tool wear with the machining force determines the burr height.

**Figure 9:**
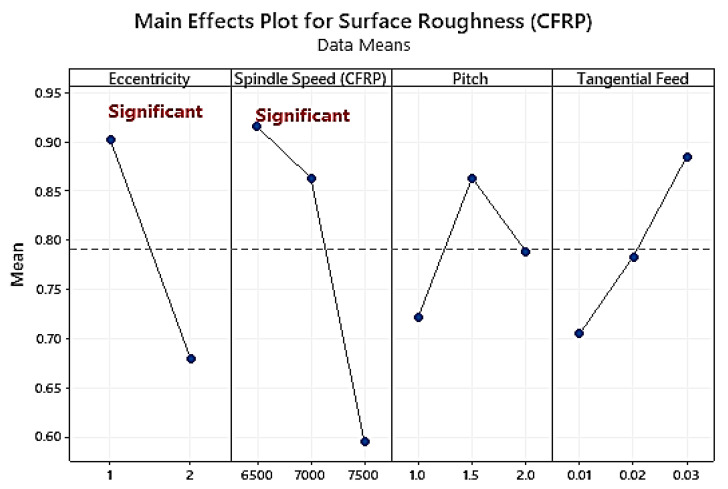
Main effects plot of average surface roughness of CFRP.

Surface roughness decreases by 33.6% with an increasing value of spindle speed [49,50] from 6500 rpm to 7500 rpm, as higher spindle speed increases the machining temperature that softens the fibres of the workpiece and makes their cutting easy by leaving fewer ups and downs in the walls of the holes (as evident from Figure 9). A similar effect was observed by Ishida et al. [51] during helical milling of CFRP stacks while working under different machining temperatures. A higher feed rate decreases the time of contact of the tool with the walls of the workpiece, so the material removal rate increases in a single pass of the tool by moving the process to instability, thus increasing the surface roughness of the holes. In this study, the increase from 1 mm to 2 mm caused an increase in the surface roughness from 0.74 to 0.79 microns in CFRP stacks.

**Figure 10:**
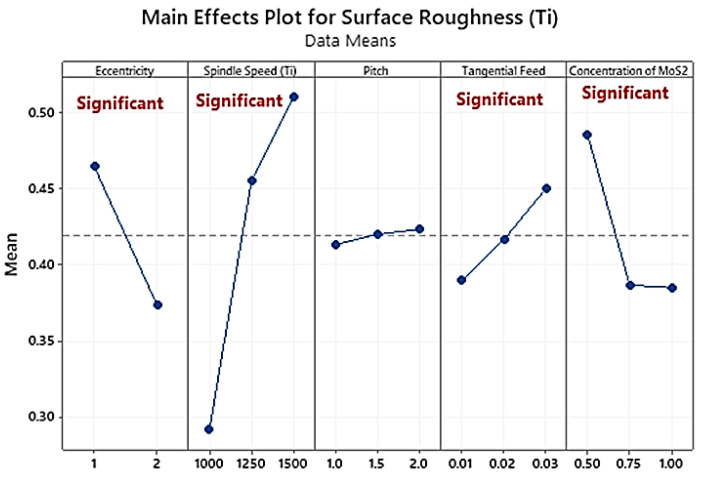
Main effects plot of average surface roughness of Ti6Al4V.

The comparison between Figure 9 and Figure 10 shows that Ti6Al4V produced a higher surface quality when compared to CFRP, regardless of the type of helical milling employed. The poor surface quality of CFRP is because it is prone to problems like matrix/fiber debonding, fibre pull-out, and cracking due to its anisotropic and nonhomogeneous characteristics [24,40].

Surface roughness is highly related to the fibre orientation in CFRP. In this study, surface roughness increased with decreasing values of cutting speed and tangential feed rate while increasing the axial feed rate value. However, the surface roughness values were very low for the multidirectional carbon fibre due to selecting suitable optimistic input parameters [14,16,52]. Surface roughness values for Ti6Al4V and CFRP were 0.68 μm and 0.92 μm, respectively. Helical milling is the most beneficial technology to achieve superior quality holes, specifically in CFRP/Ti-6Al-4V stacks [40], leaving the operators with no requirement for additional secondary processing.

There are two essential standpoints for understanding the effect of high spindle speed during the helical milling of CFRP. The first standpoint is a minute burr degradation resulting in smaller cutting forces, followed by a fibre bundle pull-out effect attributed to the higher spindle speed during helical milling of the CFRP layer. The second standpoint is that there is another effect on the CFRP fibre bundles which is observed to be the shearing force from the tool, and hence, this shearing force resultantly ameliorates hole edge quality. Besides this, it is also discernible that the outlet hole has critically deteriorated when contrasted with the inlet hole. The Ti-6Al-4V exit chip and the region near the outlet hole in CFRP had severe friction between them. It became the reason for the previous deterioration of the hole exit. The scraping effect resulting from the exit chip in Ti-6Al-4V causes excessive burr formation in the CFRP layer as the hole outlet in CFRP closer to the CFRP/Ti-6Al-4V stack interface is more susceptible to the resultant scraping effect.

In the analysis of the variance of the Ti layer, the p-value shows that the eccentricity (PCR = 15.31%) and spindle speed (PCR = 58.62%) are highly significant parameters for surface roughness. The second most significant parameters are lubrication mode (PCR = 12.30%) and tangential feed rate (PCR = 4.43%), as shown in Table 8. Roughness values increased from holes 1 to 10 for CFRP and Ti (observed in Figure 9 and Figure 10). Surface roughness for Ti is reduced with an increasing percentage of MoS_2_ in lubricant. Roughness values conform to the literature for CFRP and Ti and the aerospace standard for roughness criterion [42,45].

### 3.3. Maximum Temperature

**Figure 11:**
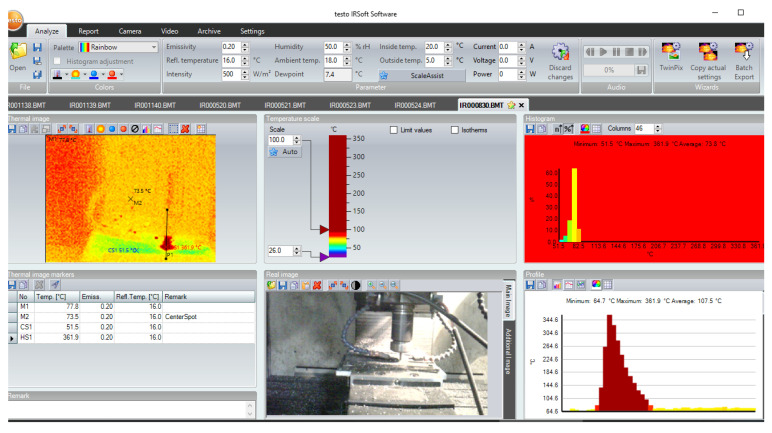
In-situ processing temperature measurement.

The cutting temperature influences the tool wear and workpiece’s surface properties during helical milling. The processing temperature changes as the stacking sequence, processing conditions, tool type/coating and cooling system change [53]. Distortion during machining of stacks might be caused by the changing temperature, which can be considered because during several studies, a thermal distortion ring on the outlet hole of CFRP has also been observed (the temperature measurement is illustrated in Figure 11). During the experimentation, resin degradation and effects like debonding were also observed in the CFRP layer as the thermal conditions got above the standard temperature. Minimal quantity lubrication is critical for the reduction of conventional cutting temperature during machining and the workpiece and tool friction coefficient,. Hence, for the machining of stacks, controlling the thermal conditions is of vital significance [21].

**Figure 12:**
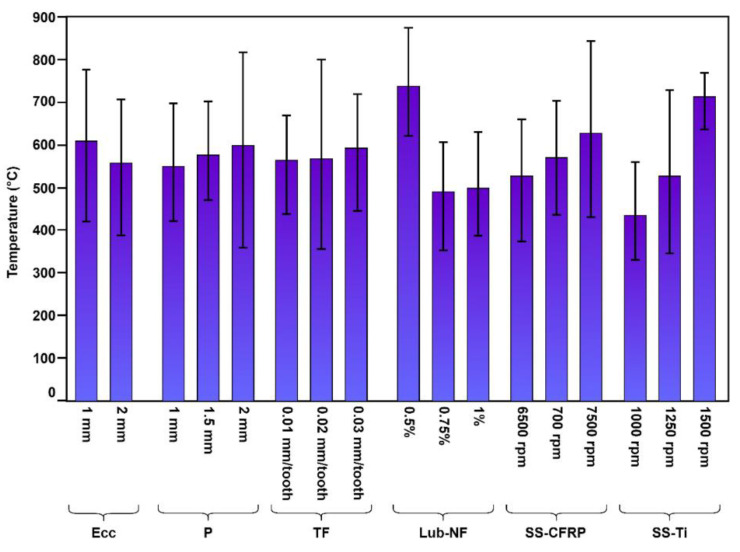
Trend plot of temperature during processing.

Critical aspects like eccentricity and speed of the spindle caused temperature rises in machining processes, as evident in the analysis of variance (Table 9). Figure 12 shows individual effects of parametric conditions with changing conditions on the temperature.

The advantage of the smaller diameter of the cutter in comparison to the milled holes in helical milling is that it allows for the cutting region to have a vent for heat exchange as the region is termed semi-closed. As an inborn characteristic, the process helps in temperature reduction [54]. Therefore, the eccentricity values have a slight variation in the decreasing trend of temperature (Figure 12). In addition to this, the sporadic nature of the helical milling also helps get down the cutting temperature. In the same way, the cutting temperature in the cutting region for conventional drilling stays high. It keeps on increasing as there is no space left for heat depletion. Other critical attributes, such as complications in chip removal, add to the cutting region’s temperature rise [42]. The maximum temperature rise during the experiment is 462 °C, which is for experiment 8 (eccentricity = 1 mm, spindle speed for CFRP and Ti = 7500 rpm and 1250 rpm, respectively, axial pitch = 2.0 mm, tangential feed = 0.02 mm/tooth, concertation of nanoparticles in MQL solution = 0.5%). The results of temperature rise are comparable to the values reported in the literature [55,56], even at low axial pitch in experimental work on helical milling of Ti. Increasing the concentration of nanoparticles in lubricant reduces the temperature as they help heat transfer [43]. Moreover, an increase in the spindle speed reflects an increase in temperature because of high cutting forces.

### 3.4. Tool Wear

The helical milling process is an intermittent operation, where the cutting edge enters and exits the workpiece several times per second with constant axial feed [57]. Therefore, tool wear phenomenon is different from other processes. Machining five holes with the tool is considered exceeding the wear limits of flank wear and edge wear, or excessive breakdown or chipping.

#### 3.4.1. Flank Wear

**Figure 13:**
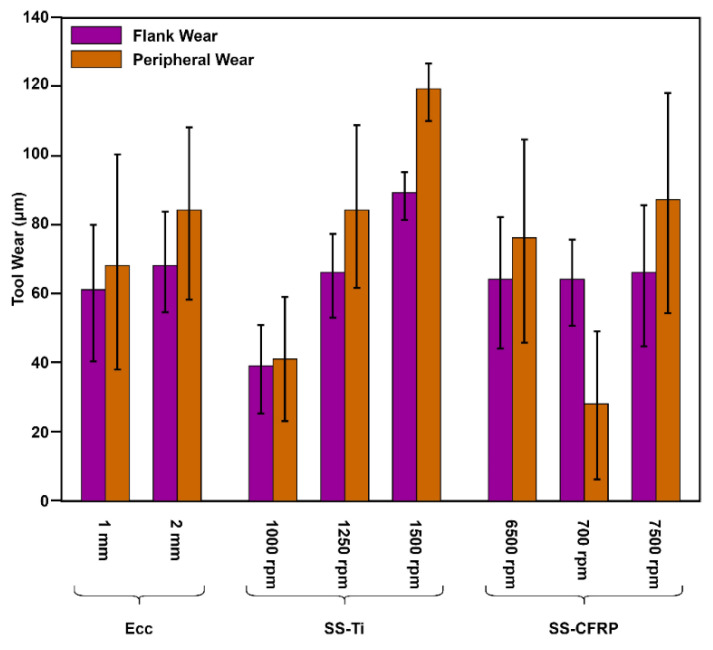
Trend plot (eccentricity, spindle speeds) of tool wear during hole-making in CFRP/Ti6Al4V.

The flank wears ~17% with eccentricity changing from 1 to 2 (Figure 13). However, there is no significant change in tool wear with the increase in spindle speed (from 6500 to 7500 rpm) in the case of CFRP. On the other hand, at a lower spindle speed of 1000 rpm, the flank wear is around 40 µm, which increases linearly by 90 µm by increasing speed up to 1500 rpm (125% increase). Similar increasing trends are observed for pitch and tangential feed. However, the flank wear substantially decreased as the concentration of MoS_2_ increased. The nano fluids influence cutting temperature, which minimises wear growth and results in longer tool life [25].

The analysis of variance of flank wear (Table 10) determines the parametric control of the process on the response. The eccentricity (2.21%), spindle speed Ti (61.16%), pitch (1.48%), tangential feed (23.37%), and lubrication (11.32%) control the output. Tool wear is remarkably dependent on the lubrication, feed, and hole-making processes in titanium.

**Figure 14:**
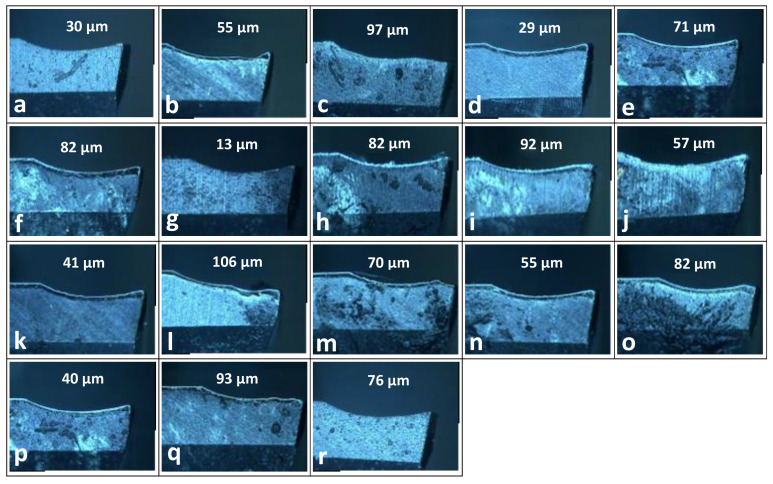
Flank wear over different parametric conditions in the experimental matrix (**a**–**r**) based on design of experiment settings.

Peripheral cutting-edge wear is higher for Experiments 3 (eccentricity = 1 mm, spindle speed for CFRP and Ti = 6500 rpm and 1500 rpm respectively, axial pitch = 2.0 mm, tangential feed = 0.03 mm/tooth, concertation of nanoparticles in MQL solution = 1%), 8 (eccentricity = 1 mm, spindle speed for CFRP and Ti = 7500 rpm and 1250 rpm respectively, axial pitch = 2.0 mm, tangential feed = 0.02 mm/tooth, concertation of nanoparticles in MQL solution = 0.5%) and 12 (eccentricity = 2 mm, spindle speed for CFRP and Ti = 6500 rpm and 1500 rpm respectively, axial pitch = 1.5 mm, tangential feed = 0.02 mm/tooth, concertation of nanoparticles in MQL solution = 0.5%). Larger values of axial pitches (2 mm and 1.5 mm) are the reasons, which are also validated by the length of peripheral wear by measurement on a CMM. Maximum flank wear is recorded for experiment 12, which is directly correlated with temperature rise and also the average burr height value of titanium. The flank wear micrographs are shown in Figure 14 at different processing conditions.

Fractography was done using a microscope, which affirms that the prominent fracture modes were abrasion wear, plastic deformation, built-up edges, and material adhesion, which is also endorsed in the literature [12]. The composition patterns of the worn-out and best tools showed that the worn tool had fewer coating components (firex), which indicated that the coating vanished from the fractured edges. In contrast, on the best-conditioned tool, the components of the coatings were there in the image showing the coating was intact. Adhesion of the particles from the lubricant surfactant and the aluminium backup plate was also there [23,58].

#### 3.4.2. Edge Wear

**Figure 15:**
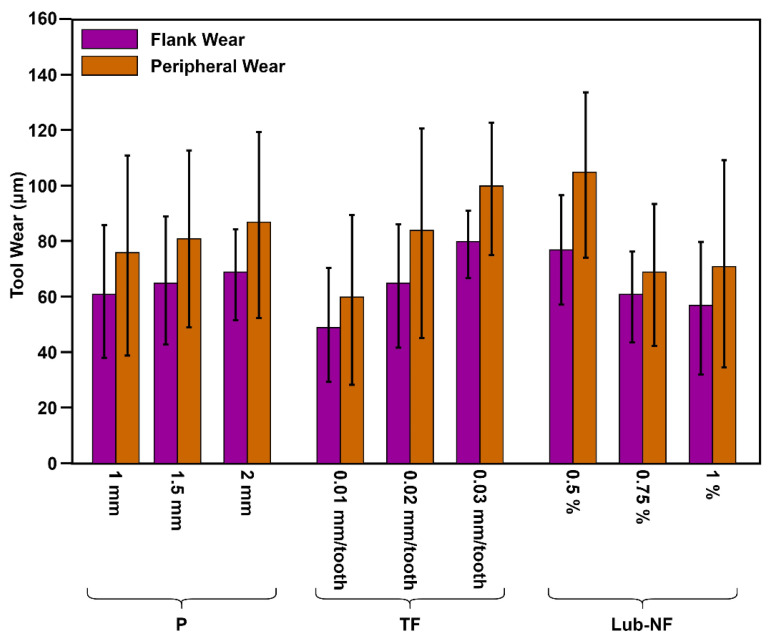
Parametric trends (pitch, tangential feed, and lubrication) of tool wear during hole-making in CFRP/Ti6Al4V.

The edge wear (Figure 13 and Figure 15) shows similar trends as flank wear, where edge wear increased ~6% with the change in eccentricity levels from 1 to 2. Similarly, at a spindle speed of 6500 rpm, the edge wear is ~75 µm which increases to ~89 µm by 7500 rpm in the case of CFRP. In the case of Ti, there is a significant change from ~40 µm to ~120 µm from 1000 rpm to 1500 rpm. The pitch and tangential feed have the same behaviour as flank wear. Moreover, the concentration of MoS_2_ decreased edge wear from ~105 µm (0.5% concentration) to ~70 µm (1% concentration).

For the case of cutting-edge wear, spindle speed Ti significantly controls (60.56%) the wear. Similarly, pitch and tangential feeds contribute equally to controlling the process (16.8%), as shown in Table 11.

### 3.5. Confirmatory Tests and Optimization

Optimisation and confirmatory tests are performed for diametric and circular errors, surface roughness, and tool wear by using Taguchi optimization with a signal-to-noise ratio with ‘smaller is better’ condition for surface roughness, tool wear, and temperature, and with ‘larger is better’ condition for hole accuracy [59].

#### 3.5.1. Hole Accuracy and Surface Roughness

In this study, hole accuracy was measured by diametric and circularity errors. Both output parameters were subjected to ‘smaller is better’ S/N ratios of both parameters and materials for optimization. After that analysis, it was found that the optimal level of parameters is the same for surface roughness in both CFRP and Ti-based materials at larger S/N ratios. It was observed that similar input parameter levels are obtained for diametric and circularity errors and surface roughness. However, the rank of different input parameters in determining optimal output parameters is different. For example, in the case of diametric error, tangential feed is the most important parameter, followed by spindle speed for CFRP and Ti6Al4V. For circularity errors, spindle speed is the most critical parameter, followed by the level of eccentricity. A higher value of eccentricity is linked with better properties. Spindle speed is the most important predictor of surface roughness in CFRP, followed by eccentricity. In contrast, for the surface roughness of Ti6Al4V, spindle speed is followed by mode of lubrication and eccentricity, respectively.

The optimised settings for CFRP, eccentricity 2 mm, spindle speed (CFRP) 7500 rpm, pitch 1 mm, and tangential feed 0.01 mm/tooth predicted the results as diametric error 6.7 μm, circularity error 1.9 μm, and surface roughness 0.36 μm. Similarly, Ti, eccentricity 2 mm, spindle speed (Ti), pitch 2 mm, tangential feed 0.01 mm/tooth, and lubrication 1% predicted diametric error 3.8 μm, circularity error 3.4 μm, and surface roughness 0.19 μm. Confirmatory experimentation was performed on the optimal settings of the parameters, and the results are shown in Table 12. For confirmatory experiments, the machining was performed on 50 holes for each experiment. The average was taken for holes 1–10, 1–20, 1–30, 1–40, and 1–50. It was found that the average value for each parameter increased with the number of holes. It is concluded that all holes drilled at optimal settings confirm the H7 standard used in the aircraft industry (max deviation = ±15 μm).

#### 3.5.2. Tool Wear

Tool wear was also optimised using the same methodology (S/N) for hole accuracy and surface roughness. All input parameters were used in the Taguchi analysis of flank and edge wear. For tool wear (both flank and edge wear), spindle speed while milling titanium is the most important input parameter. It is followed by tangential wear and mode of lubrication, respectively. It is also found that optimal levels of input parameters based on a larger S/N ratio are the same for both flank and edge wear.

**Figure 16:**
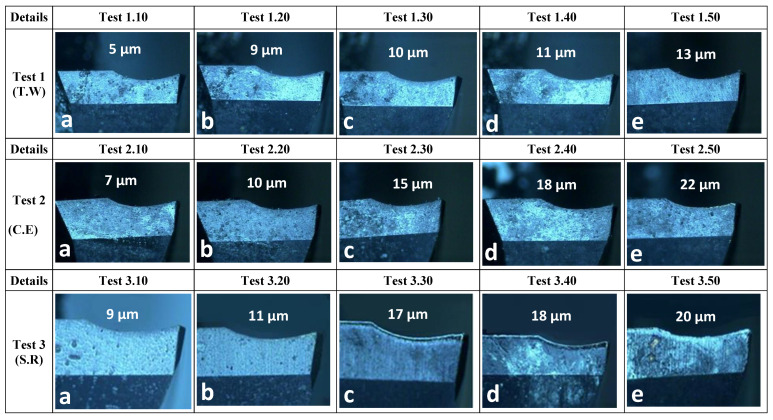
Tool wear analysis of confirmatory experiments: Test 1: SS_CFRP_ 7500 rpm, SS_Ti_ 1000 rpm, a_p_ 1 mm/rev, V_ft_ 0.01 mm/tooth, MoS_2_ 1%, ecc. 1 mm. Test 2: SS_CFRP_ 7500 rpm, SS_Ti_ 1000 rpm, a_p_ 1 mm/rev, V_ft_ 0.01 mm/tooth, MoS_2_ 0.75%, ecc. 2 mm. Test 3: SS_CFRP_ 7500 rpm, SS_Ti_ 1000 rpm, a_p_ 1 mm/rev, V_ft_ 0.01 mm/tooth, MoS_2_ 1%, ecc. 2 mm.

The optimal predicted results for eccentricity 1 mm, spindle speed (CFRP) 7500 rpm, spindle speed (Ti) 1000 rpm, pitch 1 mm, tangential feed 0.01 mm/tooth, and lubrication 1% are 9.8 µm flank wear and 5.4 µm edge wear. As confirmatory tests for hole accuracy and surface roughness, the 50 holes were drilled at optimal settings (Table 13). Tool wear is measured after every 10 holes. Similarly, the other output parameters were also measured using the methodology mentioned in Section 3.5.1. These holes also confirm the H7 standard, i.e., max deviation ± 15 μm. Micrographs showing tool wear for all confirmatory experiments are shown in Figure 16. It is evident from there that the tool wear is minimal for the optimal test parameters for tool wear.

**Figure 17:**
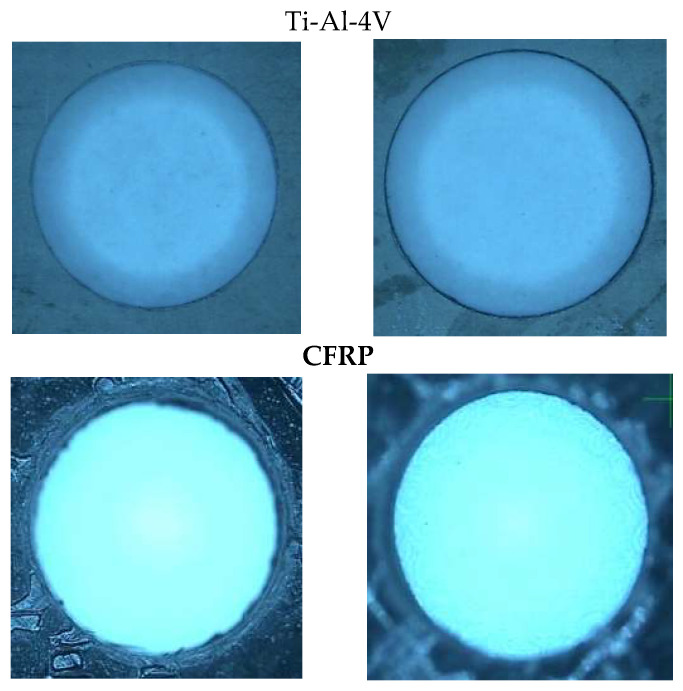
Hole interface after helical milling.

Experimental images of the hole qualities achieved at Ti-6Al-4V and CFRP layers are shown in Figure 17. The displayed holes are 10 mm in diameter with a 2 mm eccentricity.

## 4. Conclusions

This study investigated the effects of eccentricity, spindle speed, pitch, and feed rate on hole accuracy, surface roughness, maximum cutting temperature, and tool wear. Taguchi L_18_ DOE was used to design the experiments, and S/N ratios were used to analyse the results and find the optimal levels of input parameters. The following conclusions are drawn from a thorough process analysis.

- Hole accuracy was measured using diametric and circularity errors. For diametric error in CFRP, tangential feed (PCR = 40.16%) and spindle speed (PCR = 28.75%) are the most important parameters. In contrast, in the case of Ti, spindle speed (PCR = 42.84%) and eccentricity (PCR = 28.56%) are the most important parameters. For circularity error, spindle speed (PCR = 29.51% for CFRP, 30.55% for Ti) and tangential feed (PCR = 16.60 for CFRP, 43.99% for Ti) are the most significant parameters.
- Surface roughness was also analysed, and it was concluded that spindle speed (PCR = 31.73% for CFRP, 58.62% for Ti) and eccentricity (PCR = 27.32% for CFRP, 15.31% for Ti) are the most critical input parameters.
- The accuracy and geometric properties of the holes are also influenced by the maximum temperature reached during the machining process. The concentration of MoS_2_ in MQL solution is the most significant parameter in controlling the temperature (PCR = 45.76%), followed by spindle speed while machining Ti (PCR = 37.37%).
- Tool wear is also an essential parameter while machining Ti. It was found that tool wear is most affected by spindle speed while machining Ti (PCR = 61.61 for flank wear, 60.56% for edge wear), tangential feed (PCR = 27.37 for flank wear, 16.88% for edge wear), and concentration of MoS_2_ in MQL solution (PCR = 11.32% for flank wear, 16.89 for edge wear).
- Diametric and circular errors, surface roughness, and tool wear were also optimised, and 50 holes were drilled at the optimal setting. The optimal settings were found to be different for different parameters.
- All holes drilled at optimal settings for different parameters met the H7 standard. Maximum tool wear at optimal settings for diametric error was 22 μm after drilling 50 holes. The same was found to be 13μm after drilling 50 holes at optimal settings for tool wear.
- It is inferred that using MoS_2_-based MQL in helical milling of CFRP/Ti stacks results in better hole quality and surface integrity along with minimal tool wear even at remarkably high axial pitches (2 mm), which leads to a prominent decrease in machining time and enhanced tool life.

This study contributes to the knowledge of machining CFRP/Ti-6Al-4V stacks. It sheds light on the effects of different machining parameters on machining CFRP/Ti-6Al-4V stacks and is limited to studying hole quality in terms of surface roughness, hole accuracy, temperature, and tool wear. The correlation of cutting forces with the tool wear and chip morphology could be a possible extension of the work.

## Figures and Tables

**Table 1:** Prominent properties of carbon fibre [31].

Type of Fibre	Filament Count	Tensile Strength	Tensile Modulus	Density	Filament Diameter	Fibre Thickness
Plain wave 3 K, T300	3000	3530 MPa	230 GPa	1.76 g/cm^3^	7 µm	0.25 mm

**Table 2:** Experimental design to characterise parametric effects.

Sr. No.	Parameters	Levels
1	Eccentricity (mm)	1, 2
2	Spindle Speed CFRP (rpm)	6500, 7000, 7500
3	Spindle Speed Ti (rpm)	1000, 1250, 1500
4	Axial Pitch (mm)	1, 1.5, 2
5	Tangential Feed (mm/tooth)	0.01, 0.02, 0.03
6	MoS_2_ (%age)	0.5, 0.75, 1

**Table 3:** Analysis of variance of diametric error of CFRP.

Source	DF	Seq SS	Adj SS	Adj MS	F-Value	*p*-Value	Contribution
Eccentricity	1	144.5	144.5	144.50	5.19	0.039	8.41%
Spindle speed (CFRP)	1	494.1	494.1	494.08	17.74	0.001	28.75%
Tangential Feed	1	690.1	690.1	690.08	24.78	0.000	40.16%
Error	14	389.8	389.8	27.85			22.68%
Total	17	1718.5					100%

**Table 4:** Analysis of variance of the diametric error of Ti6Al4V.

Source	DF	Seq SS	Adj SS	Adj MS	F-Value	*p*-Value	Contribution
Eccentricity	1	46.72	46.72	46.722	19.99	0.001	28.56%
Spindle speed (Ti)	1	70.08	70.08	70.083	29.98	0.000	42.84%
Tangential Feed	1	14.08	14.08	14.083	6.03	0.028	8.61%
Error	14	32.72	32.72	2.337			20.00
Total	17	163.61					100%

**Table 5:** Analysis of variance of the circularity error of the CFRP.

Source	DF	Seq SS	Adj SS	Adj MS	F-Value	*p*-Value	Contribution
Spindle speed (CFRP)	1	33.33	33.33	33.33	8.22	0.012	29.51%
Tangential Feed	1	18.75	18.75	18.75	4.62	0.048	16.60%
Error	15	60.86	60.86	4.057			53.89%
Total	17	112.94					100%

**Table 6:** Analysis of variance of the circularity error of Ti.

Source	DF	Seq SS	Adj SS	Adj MS	F-Value	*p*-Value	Contribution
Eccentricity	1	12.5	12.5	12.5	1.09	0.031	11.46%
Spindle speed (Ti)	1	33.33	33.33	33.33	18.10	0.001	30.55%
Tangential Feed	1	48.00	48.00	48.00	26.07	0.000	43.99%
Error	14	15.278	15.278	1.910			14.00%
Total	17	109.11					100%

**Table 7:** Analysis of variance of surface roughness of CFRP.

Source	DF	Seq SS	Adj SS	Adj MS	F-Value	*p*-Value	Contribution
Eccentricity	1	0.2404	0.2404	0.2404	9.92	0.007	27.32%
Spindle speed (CFRP)	1	0.2760	0.2760	0.2760	11.39	0.004	31.37%
Error	15	0.3635	0.3635	0.0242			41.31%
Total	17	0.8798					100%

**Table 8:** Analysis of variance of Ti6Al4V surface roughness.

Source	DF	Seq SS	Adj SS	Adj MS	F-Value	*p*-Value	Contribution
Eccentricity	1	0.0373	0.0373	0.0373	21.29	0.000	15.31%
Spindle speed (Ti)	1	0.1430	0.1430	0.1430	81.49	0.000	58.62%
Tangential Feed	1	0.0108	0.0108	0.0108	6.15	0.028	4.43%
Lubrication	1	0.0300	0.0300	0.0300	17.09	0.001	12.30%
Error	13	0.0228	0.0228	0.0017			9.35%
Total	17	0.2439					100%

**Table 9:** Analysis of variance of maximum temperature of Ti.

Source	DF	Seq SS	Adj SS	Adj MS	F-Value	*p*-Value	Contribution
Eccentricity	1	1184	1184.2	1184.2	0.69	0.437	0.95%
Spindle Speed (CFRP)	2	7215	7215.4	3607.7	2.12	0.202	5.79%
Spindle Speed (Ti)	2	46,550	46,550.1	23,275.1	13.65	0.006	37.37%
Pitch	2	1658	1658.1	829.1	0.49	0.637	1.33%
Tangential Feed	2	719	719.4	359.7	0.21	0.816	0.58%
Lubrication	2	57,010	57,009.8	28,504.9	16.71	0.004	45.76%
Error	6	10,234	10,234.0	1705.7			8.22%
Total	17	124,571					100.00%

**Table 10:** Analysis of variance of flank wear.

Source	DF	Seq SS	Contribution	Adj SS	Adj MS	F-Value	*p*-Value
Eccentricity	1	264.5	2.21%	264.50	264.50	33.61	0.001
Spindle Speed (CFRP)	2	8.8	0.07%	8.78	4.39	0.56	0.600
Spindle speed (Ti)	2	7312.4	61.16%	7312.44	3656.22	464.56	0.000
Pitch	2	176.4	1.48%	176.44	88.22	11.21	0.009
Tangential Feed	2	2794.1	23.37%	2794.11	1397.06	177.51	0.000
Lubrication	2	1353.4	11.32%	1353.44	676.72	85.98	0.000
Error	6	47.2	0.39%	47.22	7.87		
Total	17	11,956.9	100.00%				

**Table 11:** Analysis of variance of cutting-edge peripheral wear.

Source	DF	Seq SS	Contribution	Adj SS	Adj MS	F-Value	*p*-Value
Eccentricity	1	133.4	0.44%	133.4	133.4	0.97	0.363
Spindle Speed (CFRP)	2	352.3	1.16%	352.3	176.2	1.28	0.345
Spindle speed (Ti)	2	18,386.3	60.56%	18,386.3	9193.2	66.64	0.000
Pitch	2	408.3	1.35%	408.3	204.2	1.48	0.300
Tangential feed	2	5124.0	16.88%	5124.0	2562.0	18.57	0.003
Lubrication	2	5126.3	16.89%	5126.3	2563.2	18.58	0.003
Error	6	827.8	2.73%	827.8	138.0		
Total	17	30,358.5	100.00%				

**Table 12:** Results of experiments for assessing hole accuracy and surface roughness.

Number of Holes	Diametric Error (μm)	Circularity Error (μm)	Surface Roughness (μm)	Tool Wear (μm)
10 (CFRP)	7.4	4.00	0.525	7
10 (Ti)	7	3.00	0.19	
50 (CFRP)	11.6	4.87	0.624	22
50 (Ti)	9	4.06	0.25	

**Table 13:** Output Parameters at Optimal Flank Wear.

Number of Holes	Diametric Error (μm)	Circularity Error (μm)	Surface Roughness (μm)	Tool Wear (μm)
10 (CFRP)	7.8	5	0.525	5
10 (Ti)	5	8	0.19	
50 (CFRP)	10.9	8	0.75	13
50 (Ti)	8	10	0.31	

## Data Availability

The raw or processed data required to reproduce these findings cannot be shared at this time as the data also forms part of ongoing work.
